# OTSSP167 Abrogates Mitotic Checkpoint through Inhibiting Multiple Mitotic Kinases

**DOI:** 10.1371/journal.pone.0153518

**Published:** 2016-04-15

**Authors:** Wenbin Ji, Christopher Arnst, Aaron R. Tipton, Michael E. Bekier, William R. Taylor, Tim J. Yen, Song-Tao Liu

**Affiliations:** 1 Department of Biological Sciences, University of Toledo, Toledo, Ohio, United States of America; 2 Fox Chase Cancer Center, Philadelphia, Pennsylvania, United States of America; Huazhong University of Science and Technology, CHINA

## Abstract

OTSSP167 was recently characterized as a potent inhibitor for maternal embryonic leucine zipper kinase (MELK) and is currently tested in Phase I clinical trials for solid tumors that have not responded to other treatment. Here we report that OTSSP167 abrogates the mitotic checkpoint at concentrations used to inhibit MELK. The abrogation is not recapitulated by RNAi mediated silencing of *MELK* in cells. Although OTSSP167 indeed inhibits MELK, it exhibits off-target activity against Aurora B kinase *in vitro* and in cells. Furthermore, OTSSP167 inhibits BUB1 and Haspin kinases, reducing phosphorylation at histones H2A^T120^ and H3^T3^ and causing mislocalization of Aurora B and associated chromosomal passenger complex from the centromere/kinetochore. The results suggest that OTSSP167 may have additional mechanisms of action for cancer cell killing and caution the use of OTSSP167 as a MELK specific kinase inhibitor in biochemical and cellular assays.

## Introduction

Maternal embryonic leucine zipper kinase (MELK, also called MRK38 or pEg3) is a serine/threonine protein kinase that belongs to the AMP-activated kinase (AMPK) related kinase family [1–5] (S1 Fig). The protein level and kinase activity of MELK are cell cycle regulated and peak during prometaphase [6, 7]. Previously MELK was suggested to regulate G2/M transition although there is controversy whether it functions as a negative or positive regulator for the transition [8, 9]. We have found that *MELK* is co-transcribed with a group of 64 core centromere/kinetochore components, suggesting a role in mitosis [10]. Consistently, MELK also interacts with, phosphorylates and activates transcription factor FOXM1, which drives expression of multiple mitosis regulatory proteins [11]. Furthermore, MELK has been reported to act during cytokinesis in *Xenopus* early embryos [12] and in human cancer cells [13, 14]. More interestingly, microarray profiling listed *MELK* as one of the top-ranking (#11) chromosomal instability (CIN) signature genes [15]. High level of MELK expression has been reported in cancers and cancer stem cells [4, 16, 17]. MELK is currently regarded as a promising target for novel cancer therapy, and several MELK small molecule inhibitors including OTSSP167 have been published [18–20]. However, it is still unclear whether MELK overexpression in cancer cells has any causal relationship with the CIN phenotype [17, 21–26]. The mitotic effects of MELK inhibition at molecular and cellular level remain to be fully characterized.

The mitotic checkpoint (or spindle assembly checkpoint) is an essential mechanism to maintain chromosomal stability. The checkpoint can be viewed as a specialized signal transduction mechanism that detects kinetochore-microtubule attachment defects and halts the metaphase-to-anaphase transition to prevent chromosome missegregation [27, 28]. At molecular level, signal transduction of the mitotic checkpoint leads to increase in intracellular concentration of a specific conformer of MAD2, closed MAD2 (C-MAD2), and then formation of the Mitotic Checkpoint Complex (MCC) that is composed of BUBR1, BUB3, CDC20 and C-MAD2 [29–31]. The MCC directly binds and inhibits the multi-subunit E3 ubiquitin ligase Anaphase Promoting Complex (APC/C) [31]. As APC/C activity is essential for destruction of cyclin B and securin—the prerequisites for anaphase onset, APC/C inhibition leads to mitotic arrest [32, 33].

Aurora B is the kinase component of the chromosome passenger complex (CPC, including Aurora B, INCENP, Survivin and Borealin), which participates in the regulation of chromosome alignment, mitotic checkpoint and cytokinesis [34, 35]. Accordingly, the subcellular localization of CPC shows a dynamic pattern as cells go through mitosis: mostly localized on chromosomes during prophase, enriched at inner centromeres between sister kinetochores during prometaphase with a small fraction distributed at kinetochores, and transferred to midzone and midbody as cells finish mitosis [34–37]. The inner centromere localization of CPC is mostly determined by two other kinases: BUB1 phosphorylating histone H2A at T121 creating a binding site for Sgo1, which in turn recruits Borealin [37–39]; and Haspin phosphorylating H3 at T3, to which Survivin binds [40–42]. The kinase activity of Aurora B is required for its multi-faceted mitotic functions, but seems dispensable for its inner centromere localization, as small molecule inhibitors or kinase dead Aurora B mutant did not alter its localization [43, 44].

OTSSP167 was recently characterized as a potent MELK inhibitor and is currently in Phase I clinical trials for solid tumors that have not responded to other treatment (ClinicalTrials.gov Identifier: NCT01910545) [16, 18, 45]. We found that OTSSP167 abolished the mitotic checkpoint and hereby report the results of our investigation into the phenomenon.

## Results

### MELK inhibition abrogates the mitotic checkpoint and aborts cytokinesis

**Fig 1:**
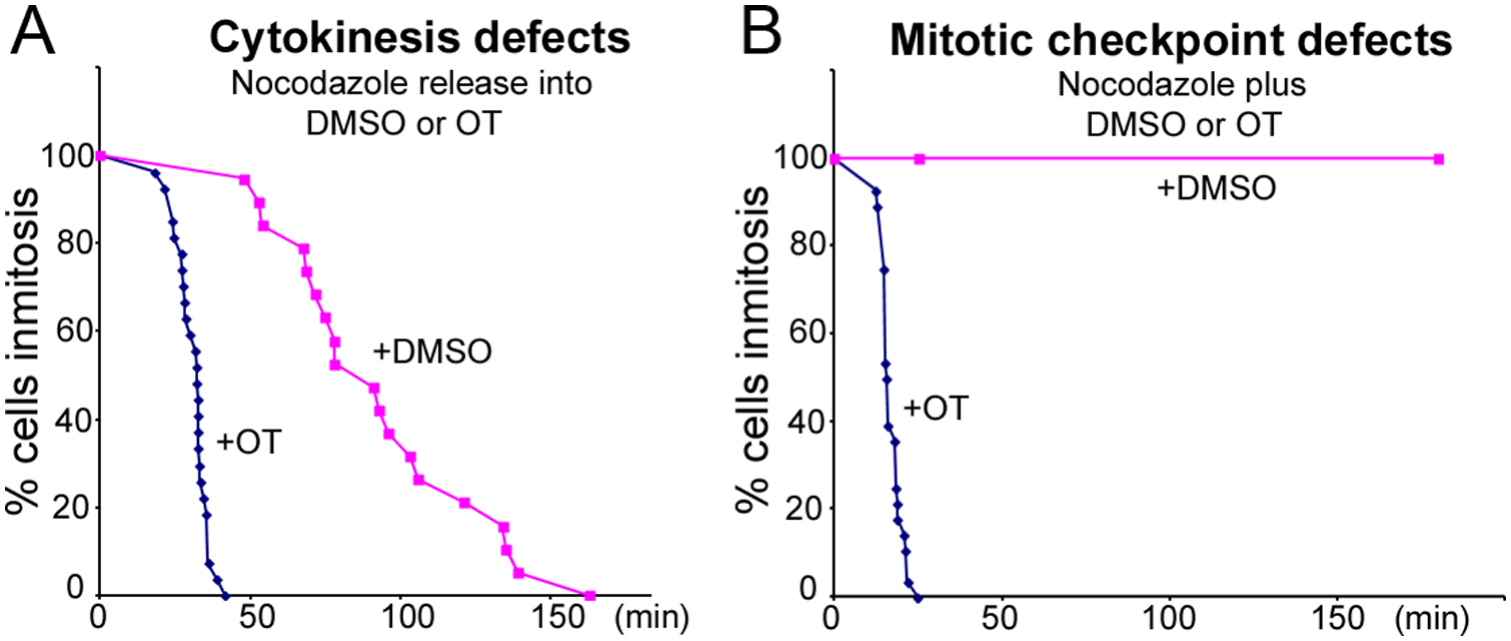
Mitotic defects caused by OTSSP167. (A) Cytokinesis failure. Nocodazole arrested mitotic MCF7-mRFP-H2A cells were washed and released either into DMSO or 100 nM OTSSP167 (OT)-containing medium. Initiation of cleavage furrow (for DMSO treated cells, n = 19) or cell flattening (for OT treated cells, n = 27) together with chromosome decondensation marked exit from mitosis. OT treated cells exited from mitosis without division. (B) Mitotic checkpoint defects. Cells arrested in nocodazole were further exposed to either DMSO or OTSSP167 (n>63). Cell flattening and chromosome decondensation marked exit from mitosis.

Previously *MELK* has been shown to transcriptionally co-express with centromere/kinetochore proteins and play a role in cytokinesis [10, 12, 46, 47]. A potent MELK inhibitor OTSSP167 has recently been reported (IC_50_ = 0.41 nM *in vitro*) [18, 45]. We tested whether OTSSP167 could interfere with mitosis. MCF7 cells blocked in mitosis by nocodazole (a microtubule depolymerizing drug) were allowed to progress synchronously through mitosis after nocodazole washout. In the control where cells were released into DMSO, the majority aligned their chromosomes, entered anaphase and proceeded through cytokinesis within 2 hours. However, cells released into OTSSP167-containing medium failed to finish cytokinesis. Instead they flattened out and entered interphase rapidly (*T*_50%_ = 32 min) without observable cleavage furrow formation (Fig 1A, S1 and S2 Videos), which is consistent with earlier suggestion that MELK may regulate cytokinesis [12, 13, 47].

The cells released from nocodazole into OTSSP167-containing medium seemed to exit from mitosis without attempting to align their chromosomes, suggesting a mitotic checkpoint override. To test this further, OTSSP167 was directly added to nocodazole arrested MCF7 cells. These cells (n = 28) also exited from mitosis rapidly (*T*_50%_ = 16 min), despite the presence of the microtubule inhibitor. As a control, 100% of nocodazole arrested cells (n = 25) stayed in mitosis when the solvent control DMSO was added (Fig 1B, S3 and S4 Videos). Similar results were observed when cells were arrested in mitosis with taxol or HeLa cells expressing mRFP-H2A were used (not shown). These cellular observations led us to conclude that OTSSP167 can abrogate the mitotic checkpoint.

### OTSSP167 disrupts MCC and MCC-APC/C interaction

**Fig 2:**
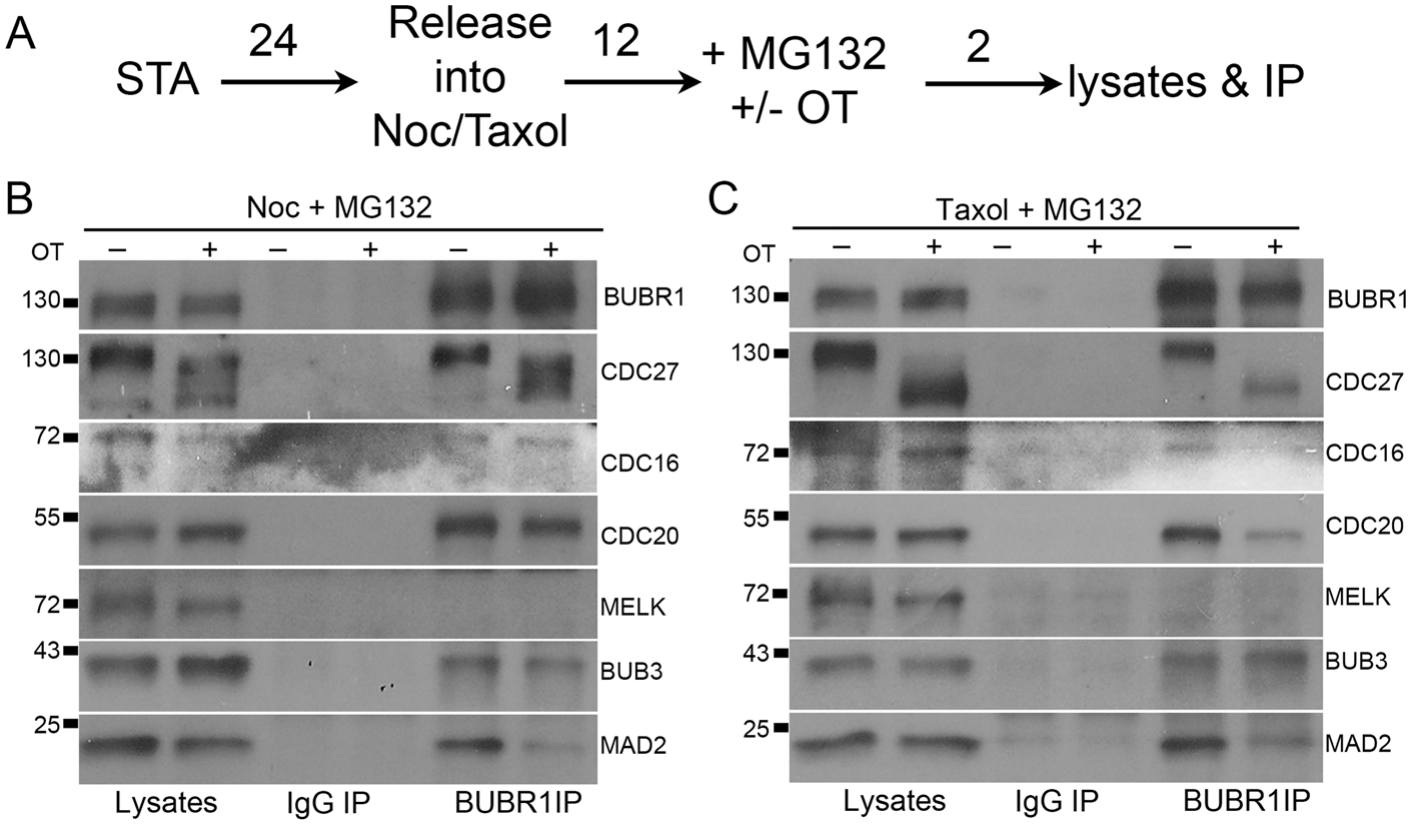
OTSSP167 causes loss of MAD2 from the MCC. (A) Outline of cell synchronization protocol. Following single thymidine arrest (STA) for 24 h (numbers above arrows indicate time in hours), HeLa cells were released into nocodazole (Noc) or taxol for 12 h and then treated with DMSO or OTSSP167 (100nM) ("+") plus MG132 (to prevent mitotic exit). After 2 h, cells were processed for lysates and immunoprecipitation (IP). (B&C) Nocodazole (B) or Taxol (C) arrested cells were treated with DMSO (“-“) or OTSSP167 (“+”) and the lysates were subjected for nonimmune rabbit IgG or BUBR1 IP and Western blot. The MCC and APC/C components were probed together with MELK. Molecular weight markers (in kDa) were labeled.

The mitotic checkpoint is maintained by MCC-mediated inhibition of the APC/C [27], therefore we tested whether OTSSP167 biochemically disrupted the MCC assembly or the MCC-APC/C interaction. As expected, immunoprecipitation (IP) using anti-BUBR1 antibody in nocodazole, DMSO plus MG132 (a proteasome inhibitor to prevent cells from exiting mitosis) treated lysates pulled down MCC subunits BUB3, CDC20 and MAD2 together with APC/C subunits CDC27 (Fig 2A and 2B, OT “-” lane in BUBR1 IP). When OTSSP167 was used instead of DMSO, a fast-migrating species of MELK appeared in the lysates, suggesting MELK dephosphorylation [7] (characterization of the immune-purified anti-MELK antibody is shown in S2 Fig). Similarly, dramatic mobility down-shift of CDC27 was also noticed in OTSSP167 treated lysates. Consistent with checkpoint override shown in Fig 1B, there was a clear reduction of MAD2 in BUBR1 IP (Fig 2B, compare MAD2 in OT “+” and “-”lanes in BUBR1 IP). We previously have shown that BUBR1:C-MAD2 (closed conformer) interaction is crucial for a functional MCC [29, 48, 49]. The result in Fig 2B suggested OTSSP167 can disrupt the MCC and thus prevent MCC from inhibiting the APC/C. Moreover, the association between MAD2 or CDC20 with BUBR1, and the interaction between MCC and APC/C (judged by CDC27 and CDC16 levels in the BUBR1 IP), were all dramatically reduced when cells arrested in mitosis by microtubule stabilizing drug taxol were further treated with OTSSP167 (Fig 2C). This is consistent with previous results that taxol induced mitotic checkpoint is thought to be weaker compared to in nocodazole treated cells [50–53]. Taken together, the results indicated that OTSSP167 compromises the mitotic checkpoint through disrupting MCC assembly and APC/C inhibition.

### MELK RNAi did not recapitulate the mitotic effects of OTSSP167

**Fig 3:**
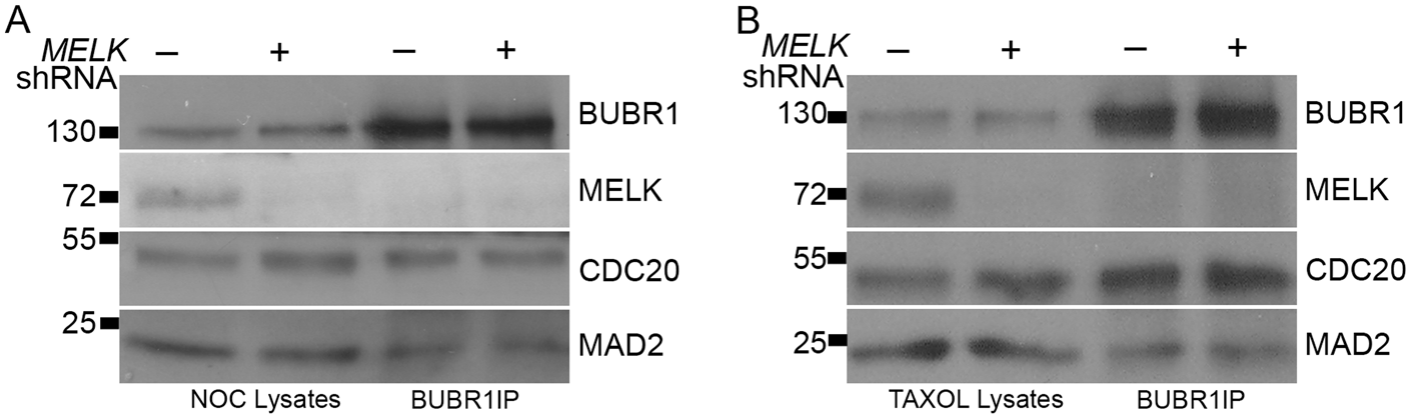
MELK knockdown does not compromise MCC assembly. HeLa cells were transfected with MELK shRNA for 24 hrs. Transfected cells were selected by puromycin while synchronized as described in Fig 2A. The nocodazole (A) and taxol (B) arrested cell lysates were subjected to IP and then probed for proteins as indicated.

*MELK* has not been implicated in mitotic checkpoint regulation before, and many kinase inhibitors especially ATP analogs have off-target effects. We therefore sought to validate the results obtained with OTSSP167 with *MELK* knockdown. Contrary to inhibition by OTSSP167, in live cell imaging experiments HeLa or MCF7 cells transfected with *MELK* shRNA arrested in mitosis after exposure to nocodazole, just as well as the vector control transfected cells. The MELK knockdown was confirmed by Western blot (Fig 3). Consistently, BUBR1 IP after MELK knockdown did not show differences in the MAD2 level whether the cells were arrested in nocodazole or taxol (Fig 3). These results suggested that the OTSSP167 effects might not be caused by *MELK* inhibition.

### OTSSP inhibits Aurora B kinase

**Fig 4:**
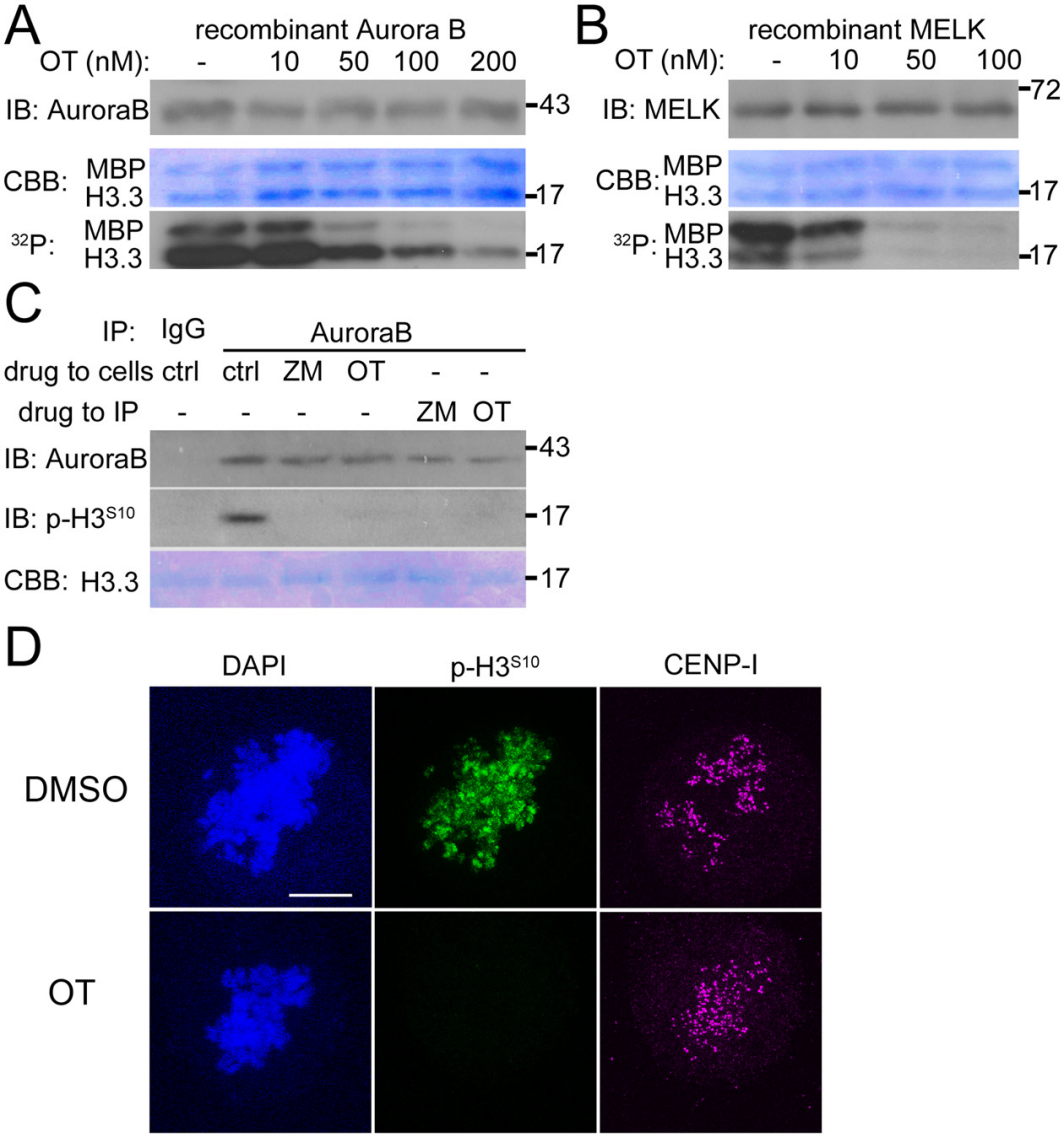
OTSSP167 inhibits Aurora B and MELK. (A) In vitro kinase assays with ~40 nM of recombinant 6×His-Aurora B/INCENP^(822–918)^ and 1 μg each of histone H3.3 and myelin basic protein (MBP). OTSSP167 (OT) was added at different concentrations (nM) with DMSO as a control (lane “-“). The samples were separated by SDS-PAGE, blotted and then processed for autoradiography (“^32^P”). The same blot was then stained shortly with Coomassie brilliant blue (CBB) to show equal loading of substrates, and probed by immunoblotting (“IB”) for Aurora B. The numbers on the right indicate molecular weight markers (kDa). (B) Similar as in (A), except that ~40 nM of recombinant GST-MELK^(1–340)^ was used. (C) HeLa cells arrested with nocodazole and MG132 were further treated with DMSO (“ctrl”), ZM447439 (ZM, 2.5 μM final concentration) or OTSSP167 (OT, 100 nM final concentration) and the lysates were used for in vitro Aurora B IP kinase assays. Alternatively the IPs from DMSO treated lysates were used in kinase assays in the presence of ZM or OT. The kinase reactions were applied to SDS-PAGE followed by Western blot. The membranes were stained for recombinant histone H3.3 by Coomassie staining before blocking. The phosphorylated H3.3 was probed with anti-phospho-H3^S10^ antibody. (D) Immunofluorescence of HeLa cells arrested with taxol and MG132 and then further treated with DMSO or OTSSP167 (OT). Anti-phospho-H3^S10^ antibody was probed to detect Aurora B activity on chromosomes. Anti-CENP-I is a marker for centromeres. DAPI stains DNA. Bar = 10 μm.

The discrepancy between results obtained between OTSSP167 and *MELK* shRNA on the mitotic checkpoint can be explained if OTSSP167 has other kinase targets. Among the known mitotic kinases, inhibition of Aurora B or Cdk1 could cause both mitotic checkpoint and cytokinesis defects. Therefore we tested whether OTSSP167 could have off-target effects on Aurora B or Cdk1 kinases. We performed *in vitro* kinase assays using recombinant 6×His-Aurora B/INCENP^(822–918)^ complex, and found that OTSSP167 inhibited Aurora B kinase activity with IC_50_ approximately at ~25 nM when using either of the substrates histone H3.3 or myelin basic protein (MBP) (Fig 4A). As a control, OTSSP167 inhibited MELK with IC_50_ at ~ 8 nM under our experimental conditions (Fig 4B). Immune complexes captured by anti-cyclin B antibodies from mitotic cell lysates were not inhibited by OTSSP167 at 100 nM, a concentration previously determined to show antitumor activities in preclinical trials[45] (data not shown).

The effect of OTSSP167 was further confirmed using Aurora B that was immunoprecipitated (IP) from mitotic cell lysates (Fig 4C). Little phospho-H3^S10^ signals was detected after the IP kinase assays performed either using lysates treated with OTSSP167 or a well-characterized Aurora B inhibitor, ZM447439 [43]. Similar results were obtained if the drugs were added directly to the IP kinase reactions. Consistent with the *in vitro* inhibition of Aurora B kinase, OTSSP167 also abolished phospho-H3^S10^ signals in mitotic cells arrested in taxol (Fig 4D). In contrast, mitotic cells after MELK knockdown still exhibited robust phospho-H3^S10^ signals (S3 Fig). The combined results supported that OTSSP167 is not only an inhibitor of MELK but also Aurora B kinase.

### OTSSP167 affects inner centromere localization of Aurora B and other CPC subunits

**Fig 5:**
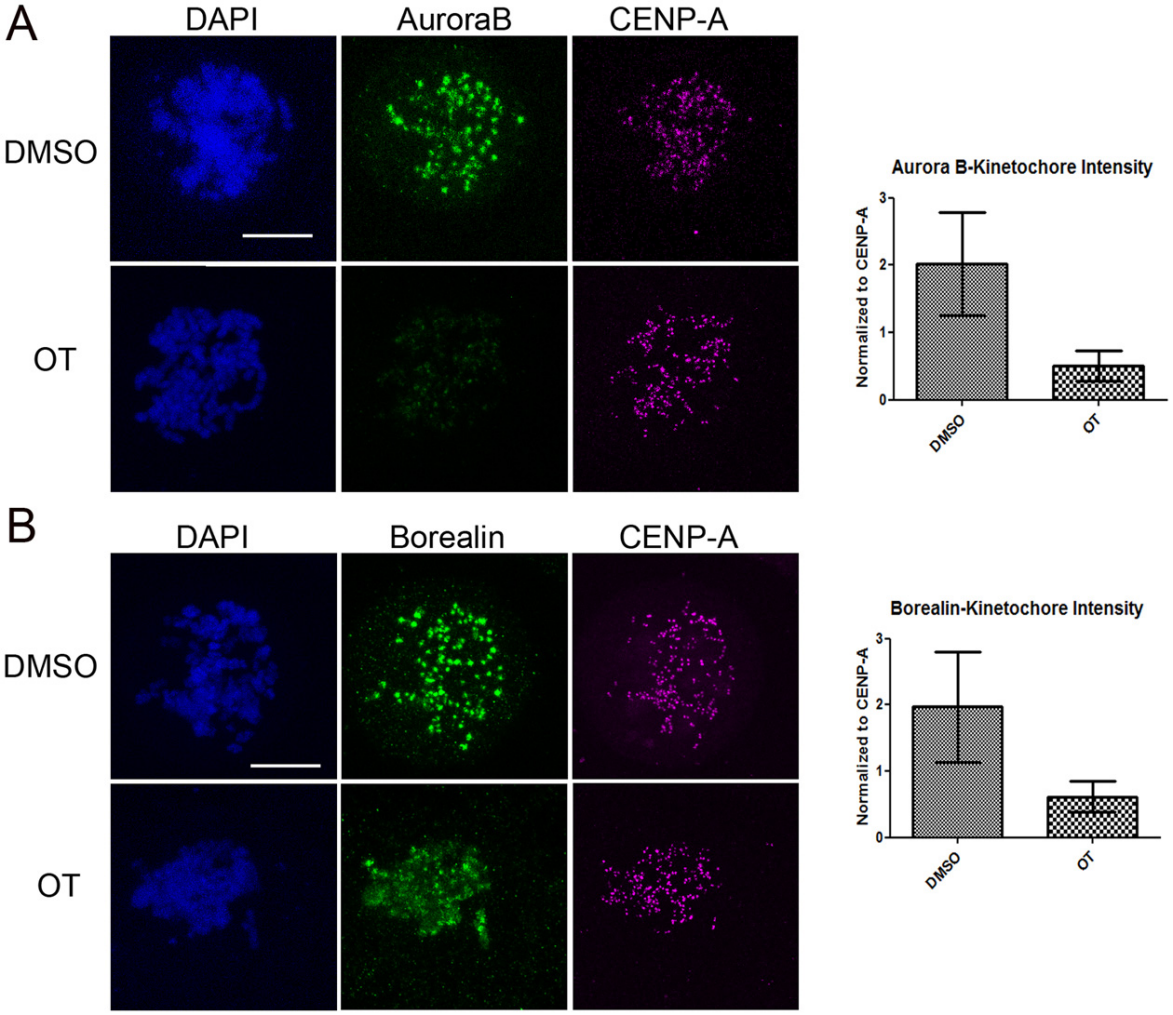
OTSSP167 mislocalizes Aurora B in mitotic cells. Taxol treated HeLa cells were further exposed to DMSO or 100 nM OTSSP167 for 2 hrs, and then fixed to probe Aurora B (A) or Borealin (B) together with CENP-A and DAPI. Maximum projections of z-stacks are shown. Bar = 10 μm. For quantification of intensities, only kinetochore pairs that fit within one z-plane were used. Relative intensities of Aurora B and Borealin were normalized to CENP-A signals and shown to the right.

While examining Aurora B activity in cells, we also stained for Aurora B and surprisingly found that it was mislocalized from inner centromeres after OTSSP167 treatment (Fig 5A). OTSSP167 treatment also caused dispersal of two other CPC subunits Borealin and Survivin from inner centromeres to spread along chromosomes (Fig 5B and data not shown), suggesting not only Aurora B but also the CPC was mislocalized.

### OTSSP167 inhibits BUB1 and Haspin kinases

Aurora B inhibition or kinase dead Aurora B was previously shown not to affect its localization at inner centromeres [43, 44, 54]. In addition, Aurora B inhibitors usually have more significant impact on taxol arrested cells but at best only caused mild mitotic checkpoint defects in nocodazole arrested cells [43, 44]. On the contrary, OTSSP167 seemed capable of efficiently driving nocodazole arrested cells out of mitosis (Fig 1B). This suggested that OTSSP167 might inhibit additional kinases other than Aurora B.

**Fig 6:**
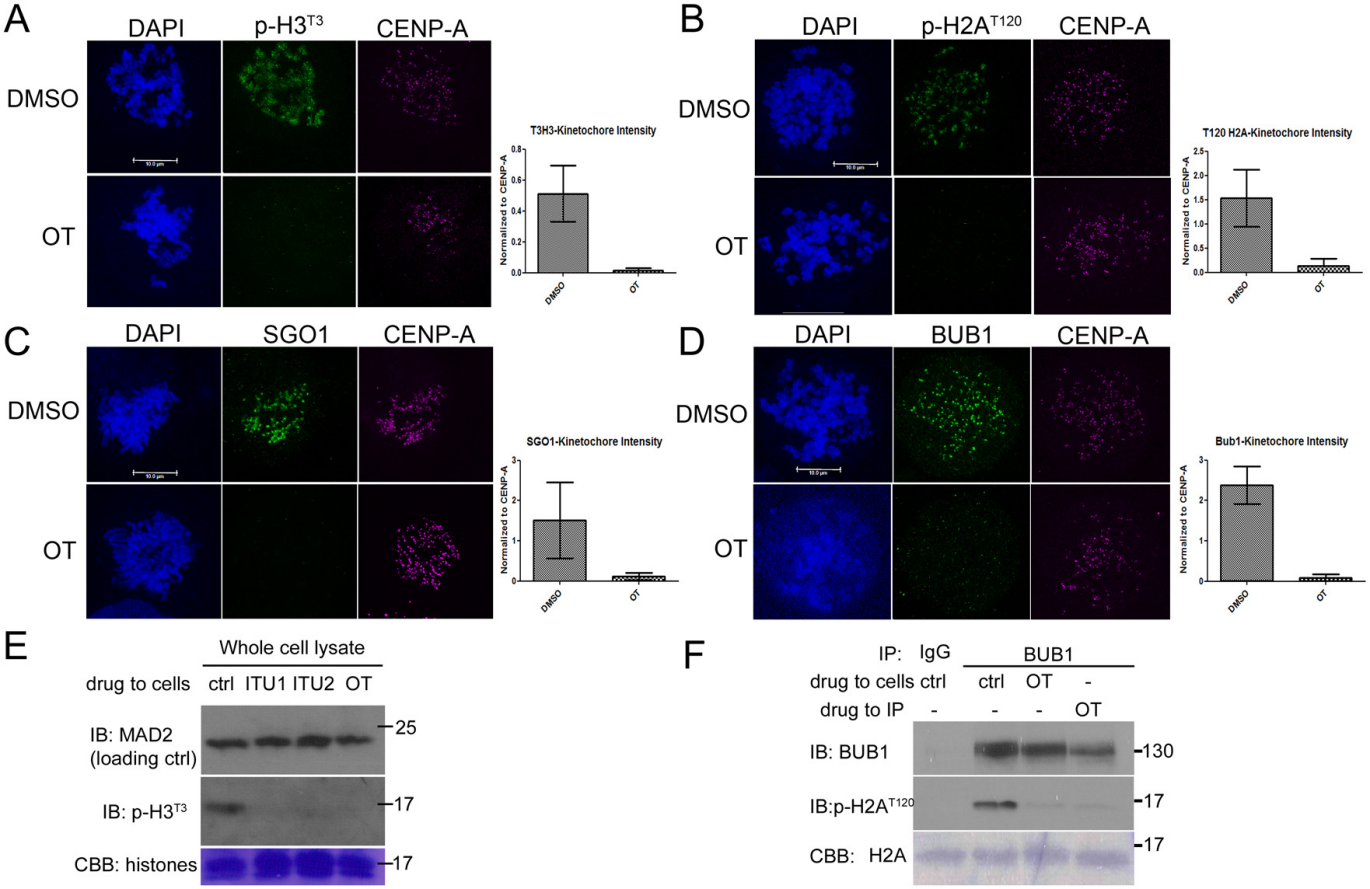
OTSSP167 inhibits Haspin and BUB1 kinases. (A-D) Taxol arrested and DMSO or OT treated HeLa cells were fixed for immunofluorescence and probed for DAPI, CENP-A and phospho-H3^T3^ (A), phospho-H2A^T120^ (B), Sgo1 (C) or BUB1 (D). Bar = 10 μm. For quantification, only kinetochore pairs that fit within one z-plane were used. Relative intensities of proteins were normalized to CENP-A signals and shown to the right. (E) HeLa cells arrested in mitosis by nocodazole and MG132 were further exposed to DMSO (“ctrl”), 5-ITU (ITU1 and ITU2 are two batches) or OTSSP167 (OT), and the whole cell lysates prepared in 1×SDS sample buffer were separated for Western blot. The membrane was stained by Coomassie blue first before being destained and probed for MAD2 or phospho-H3^T3^. (F) HeLa cells arrested with nocodazole and MG132 were further treated with DMSO (“ctrl”), or OTSSP167 (OT) and the lysates were used for in vitro BUB1 IP kinase assays. In one lane OT was also added directly to the reactions containing the IP from DMSO treated lysates. The kinase reactions were applied to SDS-PAGE followed by Western blot. The membrane was stained for recombinant histone H2A before blocking. The phosphorylated H2A was probed with anti-phospho-H2A^T120^ antibody.

The inner centromere localization of CPC depends on phosphorylation at T3 of histone H3 by Haspin kinase, and phosphorylation at T120 of histone H2A by BUB1 kinase and subsequent recruitment of Sgo1. Immunofluorescence of mitotic cells treated with OTSSP167 showed significant reduction of phospho-H3^T3^, phospho-H2A^T120^ and Sgo1 at their centromeres when compared to controls (Fig 6A–6C). Furthermore, the kinetochore localization of BUB1 itself almost disappeared after OTSSP167 treatment (Fig 6D). Immunoblotting confirmed comparable reduction of phospho-H3^T3^ signals when mitotic cells were treated either OTSSP167 or known haspin kinase inhibitor 5-ITU (Fig 6E). IP kinase assays of BUB1 found that OTSSP167 treated lysates or direct addition of OTSSP167 to the IPs abolished their respective phosphorylation of histone H2A (Fig 6F). These results indicate that Aurora B and CPC mislocalization after OTSSP167 treatment is likely caused by the loss of anchoring posttranslational modifications on centromeric histones through inhibition of BUB1 and Haspin kinases.

### OTSSP167 promotes cell cortex localization of GFP-MELK in prometaphase cells

**Fig 7:**
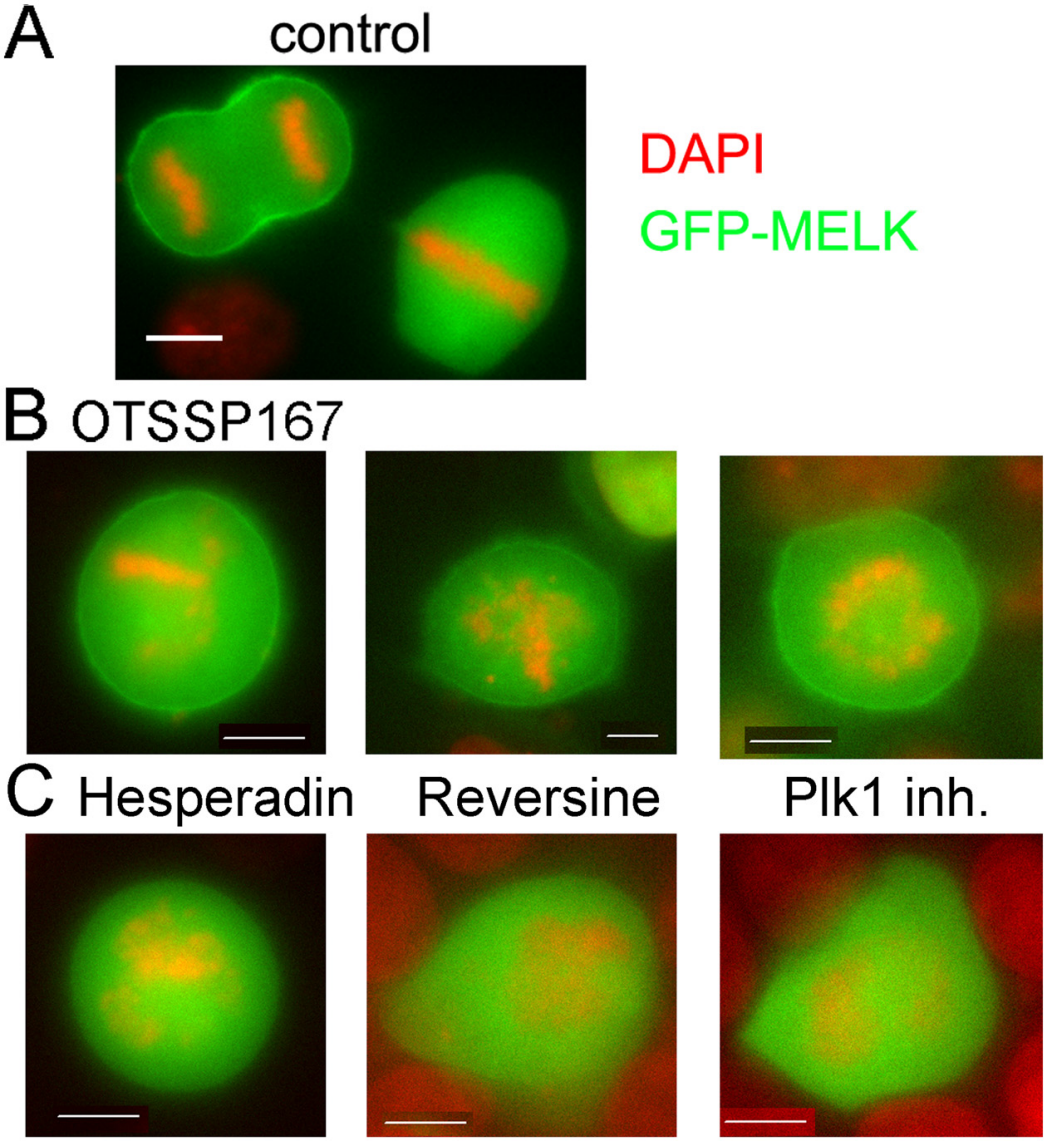
OTSSP167 causes GFP-MELK localization to cell cortex in prometaphase cells. Single-plane images of GFP-MELK transfected HeLa cells were shown in all panels. Bar = 10μm. (A) A metaphase and an anaphase cell in the same field were shown in the absence of drugs. Note the cortex association of GFP-MELK only appears obviously during anaphase. (B) Shown are three representative transfected prometaphase cells after treatment with OTSSP167 for 2 hrs. (C) GFP-MELK transfected prometaphase cells do not show cortex association after exposure to hesperadin (Aurora B inhibitor), reversine (MPS1 inhibitor) or Plk1 inhibitor III.

Consistent with earlier reports[12, 55], GFP-MELK was primarily diffuse in the cytoplasm in prometaphase cells but a fraction clearly re-localized to the cortex or cytoplasmic membrane concomitantly with the metaphase-to-anaphase transition, with some enrichment at the cleavage furrow[10, 56] (Fig 7A). Exposure of cells to OTSSP167 resulted in premature cortex association of GFP-MELK in prometaphase cells (Fig 7B). The effect seemed specific for OTSSP167, as inhibitors of other mitotic kinases such as Aurora B kinase (hesperadin), MPS1 kinase (reversine) and Plk1 kinase (inhibitor III) did not affect the timing of cortex association of GFP-MELK. The cortex association of GFP-MELK in anaphase cells was not altered by treatment of above tested kinase inhibitors.

## Discussion

Small molecule inhibitors of protein kinases have been extensively explored as targeted therapeutic agents for cancer treatment [57–59]. Currently there are over 20 kinase inhibitors approved for cancer treatment [59]. OTSSP167 was developed as a potent inhibitor of MELK and is currently in clinical trials [4, 18, 45]. OTSSP167 was reported to suppress mammosphere formation of breast cancer cells and exhibited significant tumor growth suppression in xenograft studies using breast, lung, prostate, and pancreas cancer cell lines in mice by both intravenous and oral administration [45]. The molecular mechanisms underlying the dramatic anti-proliferative effects of OTSSP167 warrant further investigation. Like many kinase inhibitors that are ATP analogs [57–59], OTSSP167 may exhibit unintended “off-target” effects against other kinases. The data presented here showed that OTSSP167 is a relatively potent inhibitor of Aurora B kinase but its dramatic impact on mitosis progression cannot be fully explained by Aurora B inhibition alone. A comprehensive kinase profiling of OTSSP167 was not available during the course of our work but off-target effects of MELK was recently cited without elaboration [60]. A web source (http://www.kinase-screen.mrc.ac.uk/kinase-inhibitors) indicated that OTSSP167 inhibited multiple kinases *in vitro*, including Aurora B and TTK/MPS1, but BUB1 and Haspin kinases were not tested. MPS1 inhibition may partially explain BUB1 loss from kinetochores as shown in Fig 6D, as recent work showed that MPS1 phosphorylation of KNL1 at the MELT motifs is essential for kinetochore recruitment of BUB1[27, 28, 61]. These results support our conclusion that OTSSP167 can compromise the mitotic checkpoint and provide an alternative to its proposed mechanism of cell killing by inhibiting MELK. Based on our findings, it may be beneficial to develop new or additional biomarkers that reflect this novel activity of OTSSP167.

MELK has become an attractive target for novel anti-cancer therapy due to its characteristic overexpression in cancer cells and cancer stem cells [4, 14–17]. Although the molecular functions of MELK remain to be fully characterized, several lines of evidence suggested it might play a role in mitosis that include G2/M transition or cytokinesis [6–13]. *MELK* knockdown showed that MELK does not have a major role in the mitotic checkpoint signaling (Fig 3). However, MELK kinase activity and protein level peak during prometaphase [6, 7] (our unpublished data), implicating some function in this stage of mitosis. One possibility is that MELK is required to coordinate cell cortex changes with chromosome segregation. As reported before [10, 56], in unperturbed mitosis a fraction of MELK is re-located to cell cortex after the metaphase-to-anaphase transition, concurring with lower activity of MELK. We observed premature cortex association of GFP-MELK in prometaphase cells when OTSSP167 was applied to cells and presumably inhibited MELK kinase activity (Fig 7). Therefore, it is possible that subcellular distribution of MELK activity is regulated by its kinase activity, and MELK activity might be important in maintaining the rounded shape of prometaphase cells. Future work will address this possibility with the help of several other recently characterized MELK small molecule inhibitors together with OTSSP167 [18–20, 62].

## Materials and Methods

### Cell culture, synchronization and drug treatment

HeLaM, a subline of HeLa [63], was maintained in DMEM with 10% fetal bovine serum at 37°C in 5% CO_2_ [29, 48, 49]. HeLaM or MCF7 cell lines stably expressing mRFP-histone H2A were cultured in the presence of 100 μg/ml Zeocin [64]. To block cells in prometaphase, HeLaM cells were treated with 2.5 mM thymidine (Sigma-Aldrich) for 24 h and then directly released into medium containing 0.2 μM (60 ng/ml) nocodazole (Sigma-Aldrich) or 10 μM taxol (Biomol International) for 12 h. Some variations of cell synchronization protocols are described in more details in figure legends. OTSSP167, a gift from Drs. Yusuke Nakamura, Takuya Tsunoda and Yo Matsuo at Onco Therapy Science Inc., was used at 100 nM [45]. Aurora B inhibitors ZM447439 (Cayman Chemical) and Hesperadin (Adooq bioscience) were used at 2.5 μM and 100 nM, respectively. The MPS1 kinase inhibitor reversine (Calbiochem), Plk1 kinase inhibitor III (Calbiochem), Haspin kinase inhibitor 5-iodotubercidin (5-ITU) (Santa Cruz), and proteasome inhibitor MG132 (Cayman Chemical) were used at 500nM, 100nM, 1 μM and 20 μM final concentrations respectively.

### DNA Constructs, RNA interference and transfection

Full length human *MELK* cDNA was amplified using primers 5’-tccagatctATGAAAGATTATGATGAACTTCTCA-3’ and 5’-caGGATCCAAGGATCCATCAATTATAC-3’, digested with *Bgl*II and *Bam*HI, and cloned into the *Bam*HI site of a home-made eGFP vector (pWS-GFP [65]) to express GFP-MELK. The *MELK* shRNA was purchased from Sigma-Aldrich with advice from Dr. Ichiro Nakano at the Ohio State University. The shRNA transfected cells were enriched by 48 hr puromycin (2μg/ml) selection starting 24 hrs post transfection. DNA transfection was carried out using TransIT-LT1 reagent (Mirus) following the manufacturer’s instructions or using polyethylenimine (PEI) following a modified protocol [66, 67]. Briefly, linear PEI (MW 25,000, from Polysciences) was dissolved in 0.2N HCl at 5 mg/ml (final pH around 1.0) for long term storage at -80°C. For transfection, the thawed PEI was neutralized to pH7.0 with NaOH and used within a month at a DNA:PEI mass ratio of 1:2.5.

### Recombinant proteins and anti-MELK antibody

A *Pst*I fragment spanning 244–651 residues of MELK from the above GFP-MELK construct was subcloned into pMALcRI vector (NEB) and expressed to make the antigen. Immunized rabbit antisera were affinity-purified from a column with immobilized antigen after cleaning up with a pre-column containing maltose binding protein expressing *E*. *coli* lysates. GST-tagged MELK^(1–340)^ and His-tagged Aurora B were expressed in *E*. *coli* BL21(DE3)-CodonPlus RIPL (Stratagene) at 37°C or 25°C and purified using GSH-agarose or Probond nickel beads (Invitrogen) [10, 29, 48]. Concentrations of recombinant proteins were determined by comparing the target band with BSA standards on Coomassie blue stained gels.

### Cell lysates, immunoblotting and immunoprecipitation

These were performed as described before [48]. A list of primary antibodies used in this study is summarized in S1 Table: Antibodies used in this study.

### *In vitro* kinase assays

Kinases were provided as recombinant proteins purified from *E*. *coli* or immunoprecipitates from mitotic cell lysates. For IP-kinase assays, the immunoprecipitates were washed twice with cell lysis buffer (1× PBS, 10% glycerol, 0.5% NP-40) supplemented with protease inhibitors (Protease Inhibitor Cocktail set III, EDTA-Free; Calbiochem) and phosphatase inhibitors (100 mM NaF, 1mM Na_3_VO_4_, 60 mM β-glycerophosphate) and twice with 1× kinase buffer (25 mM Tris-HCl, pH 7.5, 60mM ß-glycerophosphate, 10mM MgCl_2_). Myelin basic protein was purchased from Sigma and Histones H3.3 and H1_0_ were purchased from New England Labs as substrates. For kinase reactions, 4μl of 5× kinase buffer was mixed with recombinant or immunoprecipitated kinases, substrates, 5μCi ^32^P –ATP or cold ATP. H_2_O was added to make the final volume of 20 μl. The reactions were incubated at 30°C for 30 min and then terminated by adding 20 μl 2×SDS sample buffer. Samples were subjected to SDS-PAGE followed by transferring to PVDF membranes (Millipore). Phosphorylation of the substrates was visualized by autoradiography or phospho-specific antibodies.

### Immunofluorescence and live cell imaging

Cells grown on poly-lysine treated No. 1.5 coverslips were fixed in freshly prepared 3.5% paraformaldehyde containing 0.5% Triton X-100 for 10 min and then blocked with KB (10 mm Tris-HCl, pH 7.5, 150 mm NaCl, 1 mg/ml BSA) for >5 min prior to immunofluorescence. The coverslips were incubated with each primary and secondary antibody sequentially, all at 37°C for 30 minutes in a wet chamber. The secondary antibodies were the AlexaFluor series from Molecular Probes used at a 1:1000 dilution. The stained coverslips were mounted on slides using Vectashield mounting medium containing DAPI (Vector Laboratories) and imaged on a Leica TCS SP8 confocal microscope with a 63 × objective (numerical aperture = 1.40). Usually, *z*-stacks of 1.0 μm were collected and maximum projections or single focal planes were presented. For intensity measurement, kinetochore or inner centromere signals were collected from at least 10 cells and background signals were subtracted. All images for comparison were acquired and processed in the same manner. Live cell imaging of HeLaM or MCF7 cells stably expressing mRFP-H2A was performed similarly as before [49, 64] on an automated Olympus IX-81 microscope to collect phase contrast and RFP images at 3 min intervals using a 40× NA0.60 LWD (WD 2.8) objective.

## Supporting Information

S1 Fig Diagram of MELK structure. The kinase domain (11–268) is at the N-terminus, followed by a UBA domain that supports the folding and activity of the kinase domain. The functions of threonine-proline (TP) rich domain and kinase associated 1 domain (KA1) are not fully understood but may inhibit kinase activity.
(TIF)

S2 Fig Characterization of anti-MELK antibody. HeLa cells transfected with either GFP vector or GFP-MELK were lysed and the lysates were separated by SDS-PAGE and probed by anti-GFP antibody (left) then affinity-purified rabbit anti-MELK antibody (right). EndoMELK = endogenous MELK. MAD2 was also probed as a loading control. Molecular weight markers (in kDa) were labeled on the left.
(TIF)

S3 Fig MELK knockdown does not affect phosphorylation at histone H3^S10^. Immunofluorescence of HeLa cells puromycin-selected after transfection with either vector or MELK shRNA. The cells were arrested with nocodazole and MG132. Anti-phospho-H3^S10^ antibody was probed to detect Aurora B activity on chromosomes. DAPI stains DNA.
(TIF)

S1 Table Antibodies used in this study. (DOCX)

S1 Video Time lapse microscopy of a mitotic MCF7-mRFP-H2A cell released into DMSO from nocodazole arrest. The movie started ~10.0 minutes after DMSO addition due to the need to refocus. Note normal progression through mitosis with metaphase plate formation followed by cytokinesis. The time stamp marks hr:min:sec.
(MOV)

S2 Video Time lapse microscopy of a mitotic MCF7-mRFP-H2A cell released into OTSSP167 from nocodazole arrest. The movie started ~10.0 minutes after OTSSP167 addition due to the need to refocus. Note that the cell exited from mitosis by flattening out and skipping metaphase and cytokinesis. The time stamp marks hr:min:sec.
(MOV)

S3 Video Time lapse microscopy of a mitotic MCF7-mRFP-H2A cell arrested in nocodazole after further addition of DMSO. The movie started ~4.3 minutes after DMSO addition due to the need to refocus. Note that the cell remain arrested in mitosis. The time stamp marks hr:min:sec.
(MOV)

S4 Video Time lapse microscopy of a mitotic MCF7-mRFP-H2A cell arrested in nocodazole after further addition of OTSSP167. The movie started ~4.3 minutes after OTSSP167 addition due to the need to refocus. Note that the cell prematurely exited from mitosis by chromosome decondensation and flattening out in the presence of nocodazole. The time stamp marks hr:min:sec.
(MOV)
