# Supplementary figures and images for: OTSSP167 Abrogates Mitotic Checkpoint through Inhibiting Multiple Mitotic Kinases
Source: PLoS One. 2016 Apr 15;11(4):e0153518. doi: 10.1371/journal.pone.0153518 (PMC4833387; doi:10.1371/journal.pone.0153518)

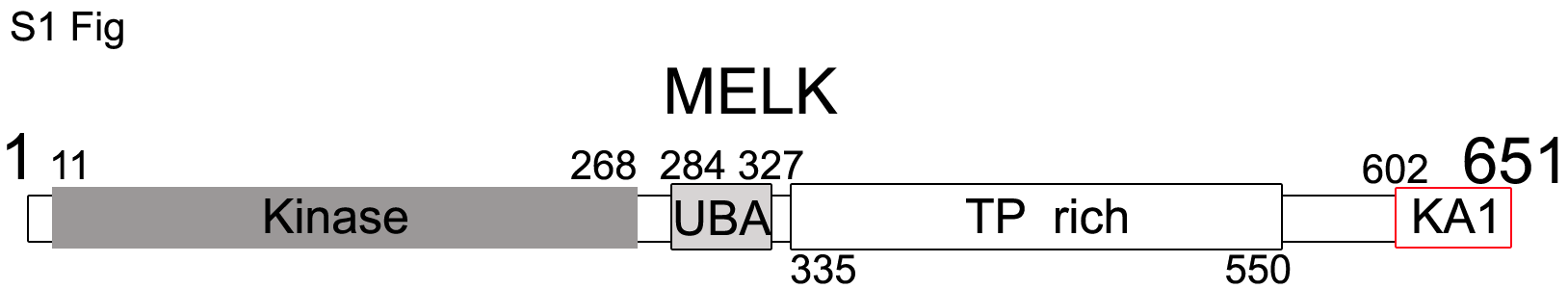

Supplement: S1 Fig — The kinase domain (11–268) is at the N-terminus, followed by a UBA domain that supports the folding and activity of the kinase domain. The functions of threonine-proline (TP) rich domain and kinase associated 1 domain (KA1) are not fully understood but may inhibit kinase activity. (TIF) [file pone.0153518.s001.tif]

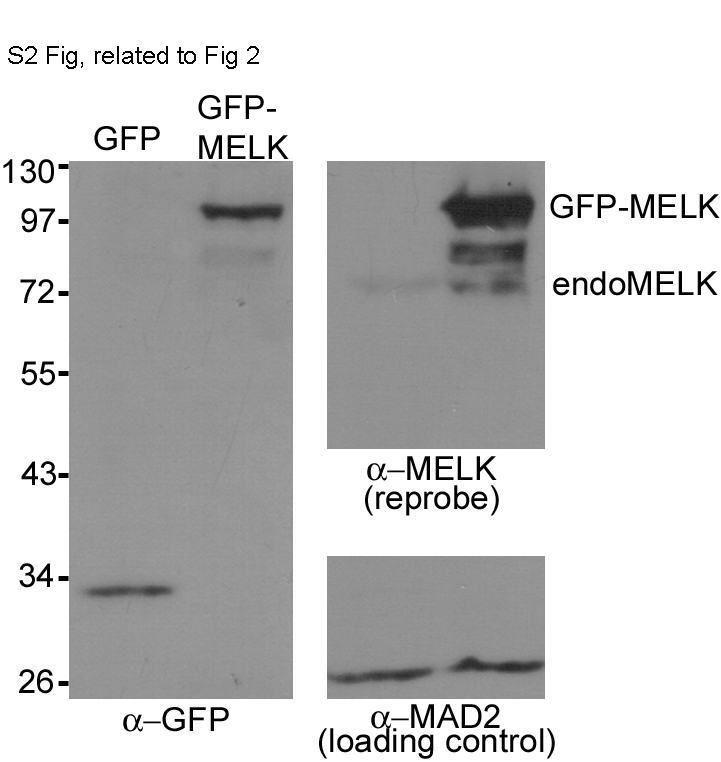

Supplement: S2 Fig — HeLa cells transfected with either GFP vector or GFP-MELK were lysed and the lysates were separated by SDS-PAGE and probed by anti-GFP antibody (left) then affinity-purified rabbit anti-MELK antibody (right). EndoMELK = endogenous MELK. MAD2 was also probed as a loading control. Molecular weight markers (in kDa) were labeled on the left. (TIF) [file pone.0153518.s002.tif]

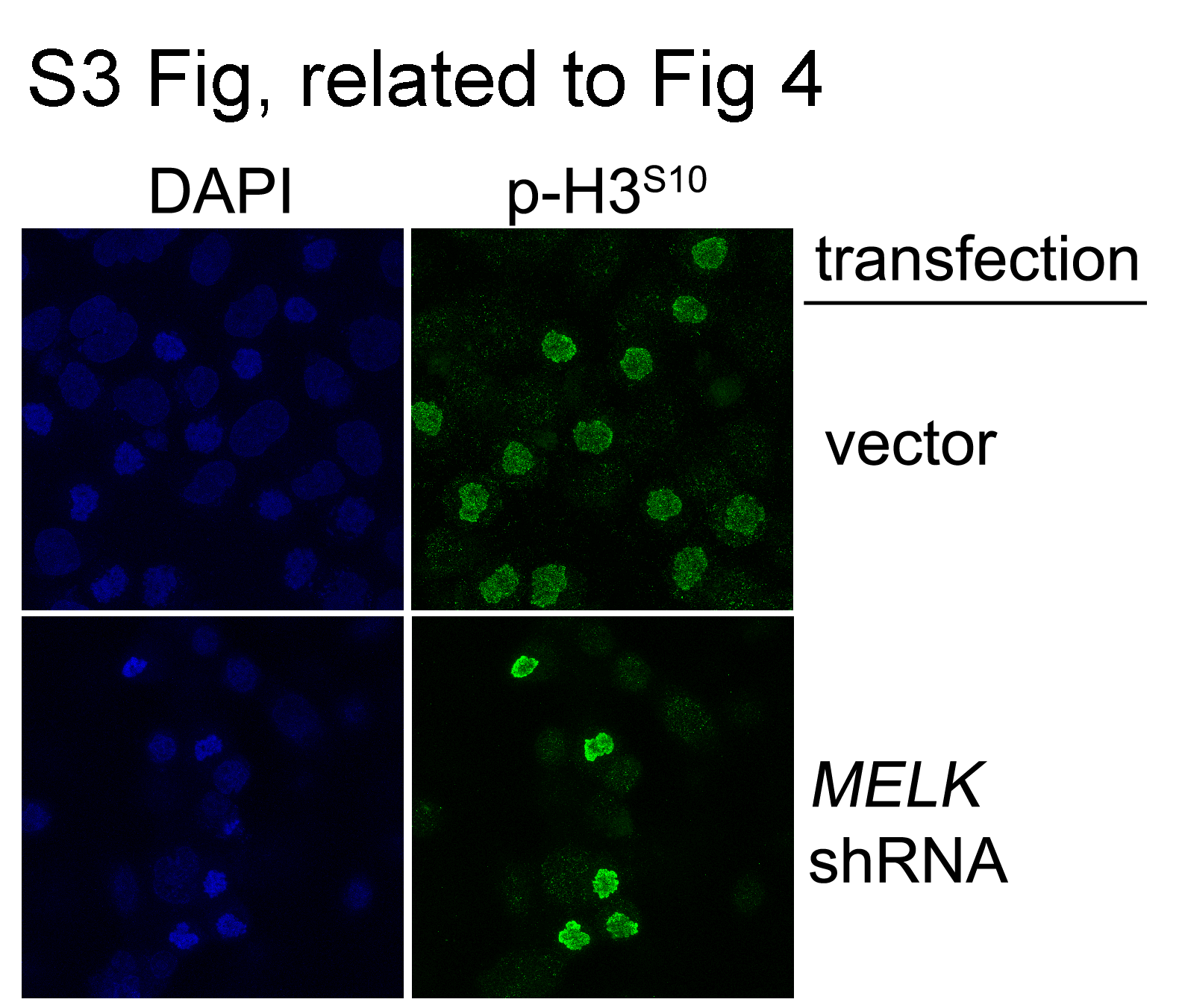

Supplement: S3 Fig — Immunofluorescence of HeLa cells puromycin-selected after transfection with either vector or MELK shRNA. The cells were arrested with nocodazole and MG132. Anti-phospho-H3S10 antibody was probed to detect Aurora B activity on chromosomes. DAPI stains DNA. (TIF) [file pone.0153518.s003.tif]
