# Supplementary material for: OTSSP167 Abrogates Mitotic Checkpoint through Inhibiting Multiple Mitotic Kinases
Source: PLoS One. 2016 Apr 15;11(4):e0153518. doi: 10.1371/journal.pone.0153518 (PMC4833387; doi:10.1371/journal.pone.0153518)
Supplement: S1 Table — (DOCX) [file pone.0153518.s004.docx]

**S1 Table. Antibodies used in this study.**

| **Target protein/tag** | **Source** | **Use** |
| --- | --- | --- |
| Aurora B | Abcam ab70238, Rabbit | IF 1:500, IP 1ug/300ug lysates |
| Borealin | [1, 2], Rabbit | IF 1:1000 |
| Bub1 | Gift From Dr. Tim Yen’s Lab, Rabbit | IF 1:250, IP 1ug/300ug lysates |
| BUBR1 | Bethyl A300-386A, Rabbit | IP 1ug/300ug lysates, IB 1:1000 |
| BUB3 | Gift from Dr. Tim Yen's Lab, Rabbit | IB 1:500 |
| CDC16 | Santa Cruz SC-6395, Goat | IB 1:200 |
| CDC20 | Santa Cruz SC-13162, Mouse | IB 1:200 |
| CDC27 | BD Transduction Laboratories 610454,  Mouse | IB 1:1000 |
| CENP-A | Mouse, [3] | IF 1:100 |
| CENP-I | Rabbit, [3] | IF 1:100 |
| GFP | Invitrogen A11122, Rabbit | IB 1:500 |
| MAD2 | Bethyl A300-301A, Rabbit | IB 1:500 |
| MELK | Prepared in this work, rabbit | IB: 1:500 |
| Phospho-Serine 10 Histone H3 | Cell Signaling Technology 97065, Mouse | IF or IB 1:1000 |
| Phospho-Threonine 3  Histone H3 | Cell Signaling Technology 97145, Rabbit | IF or IB 1:250 |
| Phospho-Threonine 120 Histone H2A | Active Motif 61196, Rabbit | IF or IB 1:250 |
| Sgo1 | Immunosoft China antibody, Rabbit | IF 1:500 |

1. Bekier ME, Mazur T, Rashid MS, Taylor WR. Borealin dimerization mediates optimal CPC checkpoint function by enhancing localization to centromeres and kinetochores. Nat Commun. 2015;6:6775.

2. Kaur H, Stiff AC, Date DA, Taylor WR. Analysis of mitotic phosphorylation of borealin. BMC Cell Biol. 2007;8:5.

3. Liu ST, Hittle JC, Jablonski SA, Campbell MS, Yoda K, Yen TJ. Human CENP-I specifies localization of CENP-F, MAD1 and MAD2 to kinetochores and is essential for mitosis. Nature cell biology. 2003;5(4):341-5.
